# The feasibility of eliminating podoconiosis

**DOI:** 10.2471/BLT.14.150276

**Published:** 2015-08-21

**Authors:** Kebede Deribe, Samuel Wanji, Oumer Shafi, Edrida M Tukahebwa, Irenee Umulisa, David H Molyneux, Gail Davey

**Affiliations:** a Brighton and Sussex Medical School, Falmer Campus, Brighton, BN1 9PX, England.; b Department of Biochemistry and Microbiology, University of Buea, Buea, Cameroon.; c Federal Ministry of Health, Addis Ababa, Ethiopia.; d Vector Control Division, Ministry of Health, Kampala, Uganda.; e Malaria and Other Parasitic Diseases Division, Rwanda Biomedical Center, Kigali, Rwanda.; f Lymphatic Filariasis Support Centre, Liverpool School of Tropical Medicine, Liverpool, England.

## Abstract

Podoconiosis is an inflammatory disease caused by prolonged contact with irritant minerals in soil. Major symptoms include swelling of the lower limb (lymphoedema) and acute pain. The disease has major social and economic consequences through stigma and loss of productivity. In the last five years there has been good progress in podoconiosis research and control. Addressing poverty at household level and infrastructure development such as roads, water and urbanization can all help to reduce podoconiosis incidence. Specific control methods include the use of footwear, regular foot hygiene and floor coverings. Secondary and tertiary prevention are based on the management of the lymphoedema-related morbidity and include foot hygiene, foot care, wound care, compression, exercises, elevation of the legs and treatment of acute infections. Certain endemic countries are taking the initiative to include podoconiosis in their national plans for the control of neglected tropical diseases and to scale up interventions against the disease. Advocacy is needed for provision of shoes as a health intervention. We suggest case definitions and elimination targets as a starting point for elimination of the disease.

## Résumé

La podoconiose est une maladie inflammatoire provoquée par un contact prolongé avec des minéraux irritants présents dans le sol. Les principaux symptômes incluent un gonflement des membres inférieurs (lymphœdème) et une douleur aiguë. Cette maladie a d'importantes conséquences sociales et économiques, car elle entraîne stigmatisation et perte de productivité. Ces cinq dernières années ont été marquées par des progrès satisfaisants en matière de recherche sur la podoconiose et de contrôle de cette maladie. Combattre la pauvreté au niveau des foyers et développer des infrastructures routières, d'approvisionnement en eau et urbaines peut contribuer à réduire son incidence. Les méthodes de lutte contre la podoconiose incluent le port de chaussures, une bonne hygiène des pieds et des revêtements de sol. La prévention secondaire et la prévention tertiaire reposent sur la gestion de la morbidité liée aux lymphœdèmes et passent par l'hygiène des pieds, les soins des pieds, les soins des plaies, la compression, des exercices, l'élévation des jambes et le traitement des infections aigües. Certains pays où la podoconiose est endémique ont pris l'initiative de l'inclure dans leur plan national pour la lutte contre les maladies tropicales négligées et de multiplier les interventions contre cette maladie. Des actions de sensibilisation sont nécessaires en vue de la distribution de chaussures comme mesure sanitaire. Nous recommandons de préciser les définitions de cas et les objectifs d'élimination pour servir de point de départ à l'éradication de cette maladie.

## Resumen

La podoconiosis es una enfermedad inflamatoria causada por un contacto prolongado con sustancias minerales irritantes del suelo. Los síntomas principales incluyen la hinchazón de las extremidades inferiores (linfedema) y un dolor agudo. Dicha enfermedad comporta graves consecuencias tanto en el entorno social como económico, ya que provoca estigmas y pérdida de productividad. En los últimos cinco años ha habido progresos satisfactorios en la investigación y el control de la podoconiosis. El tratamiento de la pobreza a nivel doméstico y el desarrollo de infraestructuras como carreteras, riegos y en urbanización pueden ayudar a reducir los casos de podoconiosis. Entre los métodos de control específico se encuentran el uso de calzado, una buena higiene de los pies y recubrimientos para el suelo. La prevención secundaria y terciaria se basa en la gestión de la morbilidad relacionada con el linfedema, esto incluye una buena higiene de los pies y el cuidado de los mismos, la cura de las lesiones, ejercicios de compresión, elevar las piernas y tratar las infecciones agudas. Algunos países endémicos han tomado la iniciativa de incluir la podoconiosis en sus planes nacionales de control sobre enfermedades tropicales desatendidas y aumentar el número de intervenciones para afrontar dicha enfermedad. Se necesita ayuda para suministrar calzado en clave de intervención sanitaria. Nuestra sugerencia para abolir la enfermedad es empezar por definir los casos y eliminar los obstáculos.

## ملخص

إن داء الفيل مرض تصحبه التهابات ويحدث نتيجة التعرض لفترة طويلة للمواد المعدنية المهيجة الموجودة بالتربة. وتشمل الأعراض الرئيسية لهذا المرض تورم الجزء السفلي من الأطراف (الوذمة اللمفية) والشعور بألم حاد. ويترتب على هذا المرض آثار اجتماعية واقتصادية كبيرة تتمثل في الحرج الاجتماعي وفقدان القدرة على الإنتاجية. وشهدت السنوات الخمسة الأخيرة درجة جيدة من التقدم في الأبحاث المتعلقة بمرض داء الفيل والسيطرة عليه. وإن مواجهة مشكلات الفقر على المستوى الأسري وتطوير البنية التحتية مثل تطوير الطرق، وموارد المياه، والعمران الحضري قد يساعد جميعه على خفض نسبة الإصابة بمرض داء الفيل. وتشمل بعض الطرق المحددة للسيطرة على المرض: استخدام الأحذية، والحفاظ على نظافة القدمين بانتظام، والحرص على استعمال المفروشات على الأرضيات. ويتوقف منع المرض على المستوى الثانوي وفوق الثانوي على إدارة مرض الوذمة اللمفية - الاعتلال المرتبط بذلك - وتشمل الحفاظ على نظافة القدمين، والاهتمام بالعناية بالقدمين، والعناية بالجروح، والضغط على منطقة التورم، وممارسة التمارين الرياضية، ورفع الساقين، وعلاج حالات الإصابة الحادة بالعدوى. وتقوم دول معينة يتوطن بها هذا المرض، بالمبادرة لتضمين مرض داء الفيل في الخطط القومية لديها، للسيطرة على الأمراض المدارية التي لا تلقى الاهتمام اللازم ولزيادة حجم التدخلات لمجابهة ذلك المرض. وتتطلب جهود الدعم توفير الأحذية كإجراء للحفاظ على الصحة. ونقترح وضع تعريف للحالات وأهداف للقضاء عليها كنقطة انطلاق للقضاء على ذلك المرض.

## 摘要

象皮病是一种由于长时间接触土壤中的刺激性矿物质而引发的炎症性疾病。 主要症状包括下肢肿胀（淋巴水肿）和剧烈疼痛。 由于患者受到歧视和降低生产力，该疾病造成重大的社会和经济影响。 过去 5 年中，象皮病研究和控制已取得很大进展。 消除贫困家庭和发展基础设施，例如道路、供水和城市化，都可以帮助降低象皮病的发病率。 具体的控制方法包括穿鞋穿袜、注意常规的脚部卫生和添加地板覆盖物。 二级和三级预防基于对淋巴水肿相关疾病发病率的管理，包括脚部卫生、脚部护理、伤口护理、加压疗法、运动、抬腿和急性感染治疗。 某些象皮病盛行的国家主动将象皮病纳入他们的国家规划，以控制这种被忽视的热带疾病，增强对疾病的干预措施。 作为一项卫生干预措施，还需要提倡鞋类的供给。 我们建议将病例定义和消除目标作为消除该疾病的起点。

## Резюме

Подокониоз — это воспалительное заболевание, вызванное длительным контактом с раздражающими минеральными частицами в почве. Основные симптомы включают отек нижних конечностей (лимфостаз) и острую боль. Заболевание имеет серьезные социальные и экономические последствия из-за стигматизации больных и потери ими трудоспособности. За последние пять лет был достигнут значительный прогресс в исследованиях подокониоза и в вопросах его контроля. Снижению частоты заболевания подокониозом помогают такие меры, как помощь бедным семьям, развитие инфраструктуры, например дорог, а также улучшение водоснабжения и урбанизация. Конкретные меры предотвращения заболевания состоят в ношении обуви, регулярной гигиене стоп и напольных покрытий в жилищах. Вторичная и третичная профилактика основаны на лечении заболеваний, сопутствующих лимфатическому отеку. Сюда входят гигиена ног, уход за стопами, лечение ран, давящие повязки, специальные упражнения, расположение ног в приподнятом положении и лечение острых инфекционных заболеваний. В определенных странах, эндемичных по подокониозу, предпринимаются инициативы по включению этого заболевания в национальные планы по контролю над тропическими болезнями, которым уделяется недостаточно внимания, а также инициативы, направленные на расширение масштаба действий, направленных на борьбу с подокониозом. Необходимо привлечь внимание правозащитников к вопросу о предоставлении населению обуви как средства медицинского вмешательства. На первых этапах мы предполагаем обозначить цели и поставить задачи по борьбе с этим заболеванием.

## Introduction

In 2012, the World Health Organization (WHO) published targets for the elimination of neglected tropical diseases or reductions in their impact to levels at which they are no longer considered public-health problems (Table 1).^1^^, ^^2^ Elimination is an attractive, motivating and powerful concept that attracts bold thinkers, increases political commitment, mobilizes donors and resources, encourages innovations and motivates health workers.^4^ It also encourages service expansion and provides access to hard-to-reach communities. Some researchers and policy-makers argue that elimination programmes may not be cost effective, may divert resources from other priorities and weaken or even destroy other disease control programmes.^5^^–^^7^ However, elimination is an attractive investment because – if the elimination effort is successful – any time-limited surge in spending should lead to long-term savings.^8^ Some definitions of important terms used by WHO in this context are listed in Box 1.

**Table 1:** Neglected tropical diseases and targets for their elimination set by the World Health Organization

Target year, disease^a^	Target	Target source
**2015**		
Chagas disease	Interruption of serological – i.e. transfusion-related – transmission in all endemic countries in Latin America	WHA resolution WHA63.20 (2010)
Dracunculiasis	Eradication, with country-by-country certification – of the elimination of transmission – by the International Commission for the Certification of Dracunculiasis Eradication	WHA resolutions WHA44.5 (1991) and WHA57.9 (2004)
Human African trypanosomiasis	Elimination from 80% of foci in selected countries	Uniting to Combat NTD (2015)^1^
Onchocerciasis in Africa	Elimination as a public health and socioeconomic problem	WHA resolution WHA47.32 (1994)
Onchocerciasis in Latin America	Elimination of the disease as a public-health problem – i.e. elimination of morbidity – and, where feasible, the elimination of *Onchocerca volvulus* transmission	PAHO resolution 14.35 (1991)
Rabies	Elimination from Latin America of human rabies transmitted by dogs, with zero cases reported to the PAHO-coordinated Epidemiological Surveillance System for Rabies	Resolution 19 of the 49th Directing Council of PAHO (2009)
Schistosomiasis	Elimination, as a public-health problem, from the Caribbean, Indonesia, WHO Eastern Mediterranean Region and areas close to the Mekong river	WHO NTD road map (2012)^2^
Yaws^b^	Elimination, with zero reporting of cases following high-quality case searches validated by independent appraisals	WHA resolution WHA31.58 (1978)
**2020**		
Blinding trachoma	Elimination as a blinding disease	WHA resolution WHA51.11 (1998)
Chagas disease	Interruption of vector- and transfusion-related transmission in all endemic countries in Latin America	WHA resolution WHA51.14 (1998)
Human African trypanosomiasis	Elimination as a public-health problem – i.e. the detection of less than one case per 10 000 inhabitants in at least 90% of endemic foci and the total number of African cases reported annually reduced below 2000	WHO meeting (2012)^3^
Leprosy	Elimination as a public-health problem – i.e. reduction in incidence in every country to less than one case per 10 000 population	WHA resolution WHA44.9 (1991)
Lymphatic filariasis	Elimination of the disease as a public-health problem and the interruption of transmission of the causative parasites^c^	WHA resolution WHA50.29 (1997) and WHO RCEM resolution EM/RC47/R.11 (2002)
Rabies	Elimination from WHO South-East Asia and Western Pacific Regions of human rabies transmitted by dogs – defined as the absence of any human rabies case following a bite or other exposure to an indigenous dog for a period of 2 years in an area where (i) circulation of dog rabies virus between dogs has been stopped by immunization and other means and (ii) an effective system for human and dog rabies surveillance and diagnosis is in place	WHO ROSEA (2012)
Schistosomiasis	Elimination, as a public-health problem, from WHO American and Western Pacific Regions and from selected countries in Africa	WHO NTD road map (2012)^2^
Visceral leishmaniasis	Reduction of annual incidence in every sub-district of India to less than one case per 10 000 population	WHO NTD road map (2012)^2^ and WHO ROSEA (2012)
Yaws	Eradication – defined as the absence of new cases for a continuous period of 3 years, supported by the absence of evidence of transmission in serosurveys among children aged < 5 years	WHA resolution WHA31.58 (1978)

NTD: neglected tropical diseases; PAHO: Pan American Health Organization; RCEM; Regional Committee for the Eastern Mediterranean; ROSEA: Regional Office for South-East Asia; WHA: World Health Assembly; WHO: World Health Organization.

^a^ Updating of target dates means that the target years given in several World Health Assembly resolutions do not match those shown here.

^b^ Endemic treponematoses.

^c^ In 2015, the working goal is a 5-year cumulative incidence of less than one case per 1000 among children who are aged 6–10 years, who were born after the initiation of mass drug administrations and lived in areas covered by such administrations for at least 5 years.

Box 1 **World Health Organization’s definition of terms used in elimination and eradication efforts^9^^,^^10^**

**Control**

Reduction of disease incidence, prevalence, morbidity or mortality to a locally acceptable level as a result of deliberate efforts – with continued intervention measures required to maintain the reduction.

**Elimination of disease**

Reduction to zero of the incidence of a specified disease in a defined geographical area as a result of deliberate efforts – with continued intervention measures required to maintain the elimination.

**Elimination of infection**

Reduction to zero of the incidence of infection caused by a specified agent in a defined geographical area as a result of deliberate efforts – with continued measures required to prevent the re-establishment of transmission.

**Elimination as a public-health problem**

Control of the manifestations of a disease – at an arbitrarily defined qualitative or quantitative level – so that the disease is no longer considered a public-health problem.

**Eradication**

Permanent reduction to zero of the worldwide incidence of infection caused by a specific agent as a result of deliberate efforts – with intervention measures no longer needed.

**Extinction**

Complete removal of the specific infectious agent so that it no longer exists in nature or the laboratory.

Podoconiosis (non-filarial elephantiasis) – an inflammatory disease caused by prolonged contact with irritant minerals in soil – was identified by WHO as a neglected tropical disease in 2011, but no global target has been set for its elimination.^11^ In recent years, there has been remarkable progress on podoconiosis research; the commitment of endemic countries to podoconiosis control has increased and elimination is now on the global health agenda.^11^ In this paper, we describe the symptoms of the disease, its socioeconomic impact, strategies for control and the feasibility of elimination.

### Symptoms and diagnosis

At present, podoconiosis can only be diagnosed clinically from characteristic signs of the disease and the exclusion of infectious and hereditary causes of lymphoedema.^12^ The key early signs of podoconiosis are splaying of the forefoot, swelling of the foot and lower leg that disappears after overnight rest, thickening of the skin over the dorsum of the foot, and moss-like rough, warty growths on the feet.^13^^–^^15^ With time, the swelling of affected legs (lymphoedema) becomes either soft and pitting or nodular and fibrotic. Late-stage disease is characterized by fusion of the toes and joint stiffness.^13^^–^^16^ The patient’s history and the results of a physical examination and certain disease-specific tests may allow filarial elephantiasis, lymphoedema of systemic disease or leprosy to be excluded.^17^

Although there are point-of-care diagnostic tests for lymphatic filariasis, such tests are not very sensitive in detecting filarial infection among advanced cases. The absence of any point-of-care tests for the diagnosis of podoconiosis is a continued challenge, especially when considering the disease’s elimination. Until such diagnostic tests are designed, the standardization of the disease’s clinical diagnosis will remain important and will involve establishing the predictive value of each of the various signs and symptoms. Previous studies have indicated that clinical diagnosis is an accurate and workable approach in settings where podoconiosis is endemic.^18^ Similar studies now need to be conducted in settings in which lymphatic filariasis and podoconiosis may overlap. The effectiveness of clinical diagnosis in excluding other causes of lymphoedema needs to be formally evaluated.

### Socioeconomic impact

Podoconiosis has severe health, social and economic consequences.^19^ According to a study in Ethiopia, the annual economic cost of podoconiosis in an area with 1.7 million residents was more than 16 million United States dollars (US$).^19^ When extrapolated to the national population, this result indicates a corresponding cost of more than US$ 200 million. People with podoconiosis were found to lose 45% of their economically productive time because of morbidity associated with the disease.^19^ Most people with podoconiosis in Ethiopia experience an episode of acute inflammation that may be triggered by bacterial, viral or fungal infection, at least once per year.^20^^–^^22^ Such acute attacks are characterized by hot, painful and reddened swelling. Since podoconiosis patients become bedridden during such attacks, it leads to loss of productivity.

The social impact of podoconiosis is also substantial. In endemic areas of southern Ethiopia, the disease is considered to be the most stigmatizing health problem^23^^,^^24^ and affected people may be excluded from school, denied participation in local meetings, churches and mosques and excluded from marriage with unaffected individuals.^23^^–^^27^ In the same areas, most community members investigated were found to have negative, stigmatizing attitudes towards social interactions with people affected by podoconiosis.^25^ In northern Ethiopia, people with podoconiosis were found to have much lower quality-of-life scores, in all domains of quality of life, than healthy people from the same neighbourhoods.^28^

The burden of podoconiosis will be estimated for the first time in the 2015 round of the *Global burden of disease study*.^29^ Clearly-generated disability-weight measurements and the inclusion in any assessment of the multiple impacts of podoconiosis – including acute attacks – should enable reasonably accurate estimates of the numbers of disability-adjusted life years lost because of the disease.

### Control strategies

Strategies aimed at addressing poverty at household level and infrastructure development such as roads, water and urbanization can all help to reduce podoconiosis incidence.^13^ Although the disease was probably once common in Algeria, the Canary Islands, Morocco and Tunisia, it has disappeared from these areas as the result of urbanization and socioeconomic development and the consequent, almost universal, use of shoes.^13^ Specific strategies for podoconiosis control may be divided into primary, secondary and tertiary prevention.^30^ Primary prevention – i.e. the prevention of contact between feet and the minerals in the irritant soil that trigger the inflammatory process – includes the use of shoes, regular foot hygiene and floor coverings. Secondary and tertiary prevention are based on the management of the lymphoedema-related morbidity and include foot hygiene, foot care, wound care, compression, exercises, elevation of the legs and treatment of acute attacks.

The objectives of secondary and tertiary prevention are to arrest progress of early disease, reduce the frequency of acute attacks and reduce the swelling of the limbs. Surgical removal of nodules may be indicated.^31^ Management of lymphoedema can lead to modest clinical improvement and substantial improvements in quality of life.^32^ The effectiveness and cost–effectiveness of these interventions are being evaluated.^33^

Much of the available information on podoconiosis treatment and prevention comes from Ethiopia.^30^^,^^34^ Local strategies include the distribution of shoes in schools^35^ and integration with the national community-based health extension programme. In a small study in northern Ethiopia, over a third of people were willing to pay at least half of the cost of care and over 30% were willing to pay the full cost of shoes.^36^

Use of shoes also has benefits in the fight against several other neglected tropical diseases.^37^ There is likely to be synergy between the elimination of podoconiosis and the elimination of lymphatic filariasis. The latter has two pillars: (i) transmission interruption via mass drug administrations and (ii) clinical care and disability prevention.^38^ It should be relatively easy to use the second pillar for both diseases.

### Societal and political considerations

There is a growing political commitment for interventions against podoconiosis. In the absence of any relevant global strategies, several endemic countries have taken independent initiatives to address the challenges of control. For example, the Ethiopian Government identified podoconiosis as one of its eight priority neglected tropical diseases and included the disease in its 2013–2015 integrated master plan for the control of such diseases.^39^ Ethiopia and Rwanda, (personal communication available from author), have each mapped the geographical distribution of podoconiosis nationwide.^12^^,^^17^ The only potentially endemic country that is currently experiencing severe political unrest is the Democratic Republic of the Congo, where the burden of podoconiosis – and the feasibility of its elimination – have yet to be studied. Risk maps can be developed based on the available evidence of environmental factors that are predictive of podoconiosis.^40^

Since WHO included podoconiosis in its list of neglected tropical diseases, the international community has responded. The Wellcome Trust and the United States National Institutes of Health continue to be strong funders of podoconiosis research.^41^ The Big Lottery Fund (Manchester, United Kingdom) supports podoconiosis interventions financially^42^ and the TOMS® shoe company (Playa Del Rey, USA) is also an important funder of podoconiosis prevention and care – both through donations of children’s shoes and via direct financial support.^42^

### Feasibility of elimination

The elimination of podoconiosis is likely to be feasible. The fact that the disease is not infectious makes elimination easier. As discussed above, use of shoes is a practical intervention for prevention of podoconiosis. Consistent use of shoes, regular foot hygiene and covering floors are the key preventive strategies against podoconiosis. Although these measures appear simple, there are social, practical and logistical challenges that hinder their acceptance by people living in endemic areas. Financial constraints, lack of appropriate shoes for wet and dry seasons and sociocultural factors all affect the availability and use of shoes. If shoes are to be used as a health intervention, innovative approaches are needed to make them affordable. Shoes also need to be appropriate to local activities and seasonal patterns.^22^^,^^43^

## Conclusion

We have developed case definitions (Box 2) and elimination targets (Box 3) as a starting point for future efforts towards the elimination of podoconiosis. A global strategy for the elimination of this disabling disease is now needed, based on the evidence for both diagnosis and care. A clearer case for investment must be provided for funding agencies and endemic-country governments. The interventions for podoconiosis prevention and treatment are relatively simple. Restored function and improved quality of life can be achieved for people with lymphoedema after just three months of treatment.^30^^,^^32^ Promotion of shoes for podoconiosis prevention is likely to have multiple health benefits.^37^

Box 2 **Case definitions of podoconiosis**

**Suspected case**

Any lymphoedema of the lower limb of any duration.

**Probable case**

Any lymphoedema of the lower limb present for more than one year in a resident of an endemic area.

**Confirmed case**

Lymphoedema of the lower limb present for more than one year in a resident of an endemic area, for which other causes – e.g. onchocerciasis, lymphatic filariasis, leprosy, Milroy syndrome, heart failure and liver failure – have been excluded.

Box 3 **Podoconiosis elimination targets**

**Elimination from an endemic district or implementation unit**

Podoconiosis is considered to be eliminated if the prevalence of untreated podoconiosis among individuals aged ≥ 15 years is < 1%, and > 95% of lymphoedema cases are treated adequately after 10 years of programme implementation.

**Elimination from country**

Podoconiosis is considered to be eliminated when:

- prevalence of untreated podoconiosis among individuals aged ≥ 15 years, in every village sampled over a 10-year period, is < 1%;
- after 10 years of control programme implementation, the prevalence of early signs of podoconiosis among children aged 10–15 years is < 0.001%;
- protective shoes are worn by > 95% of the population in endemic districts; and
- almost all (> 95%) of the lymphoedema cases are treated adequately.

By integrating podoconiosis care into the wider context of general foot care – including for leprosy and lymphatic filariasis – it should be possible to avoid duplication of efforts and enable the available resources to be used efficiently.^38^ The continued challenge in providing clinical services at scale is a shortage of skilled health workers. In those countries where podoconiosis is endemic, most health workers lack the knowledge and practical skills needed to address the symptoms of lymphoedema.^44^ Clinical management needs to be included in these countries’ curricula for the pre- and in-service training of health-care providers. National accreditation bodies for health-care providers need to be engaged in efforts to eliminate podoconiosis, and the disease needs to be included in any continued professional development for health workers.

Access to treatment services for those in need is an important aspect of podoconiosis elimination. Clinical services are currently provided by just a few faith-based and nongovernmental organizations.^30^^,^^32^ Provision of free-of-charge or low-cost prevention and treatment, through government-led programmes, will probably be critical to any elimination effort.

We need advocacy for shoes as a health intervention. For those individuals who cannot afford to buy shoes, subsidized distribution – perhaps via collaboration with shoe companies – should be considered. The TOMS® shoe company currently provides a pair of shoes to a child at risk of podoconiosis for each pair of shoes it sells elsewhere.^45^ Extending similar collaborations to other shoe companies would be beneficial.

Finally, continued research should focus on the development of good point-of-care diagnostic tests for podoconiosis, which are needed both to detect new cases and, ultimately, to verify elimination. Although definitive diagnosis may not be a priority as elimination programmes are launched, robust, sensitive and specific diagnostic tests will certainly be needed as podoconiosis becomes rare.
